# Morniga-G, a T/Tn-Specific Lectin, Induces Leukemic Cell Death via Caspase and DR5 Receptor-Dependent Pathways

**DOI:** 10.3390/ijms20010230

**Published:** 2019-01-08

**Authors:** Guillaume Poiroux, Annick Barre, Mathias Simplicien, Sandrine Pelofy, Bruno Ségui, Els J. M. Van Damme, Pierre Rougé, Hervé Benoist

**Affiliations:** 1 Université de Toulouse, Cancer Research Center of Toulouse, INSERM UMR 1037, 2 Avenue Hubert Curien, 31037 Toulouse, France; guillaumepoiroux@gmail.com (G.P.); bruno.segui@inserm.fr (B.S.); 2 Université de Toulouse, UMR 152 PharmaDev, Université Paul Sabatier, Institut de Recherche et Développement, Faculté de Pharmacie, 35 Chemin des Maraîchers, 31062 Toulouse, France; annick.barre@univ-tlse3.fr (A.B.); simplicien.mathias@gmail.com (M.S.); sandrine.pelofy@gmail.com (S.P.); pierre.rouge@free.fr (P.R.); 3 Department of Biotechnology, Faculty of Bioscience Engineering, Ghent University, Coupure links 653, B-9000 Ghent, Belgium; ElsJM.VanDamme@UGent.be

**Keywords:** plant lectin, Morniga-G, *O*-glycosylation, T/Tn antigen, Jurkat cells, cancer cell death, apoptosis, TRAIL/DR5 pathway

## Abstract

Morniga-G, the Gal-specific black mulberry (*Morus nigra*) lectin, displays high affinity for T (CD176) and Tn (CD175) antigens, frequently expressed at the cancer cell surface. The effects of Morniga-G were investigated on a Tn-positive leukemic Jurkat cell line. The lectin, used in a concentration range between 5–20 μg/mL, induced cell death in leukemic Jurkat cells. Microscopic and cytofluorometric analyses indicated that Jurkat cell death was essentially apoptotic, associated with an increase in the ceramide content and a depolarization of the mitochondrial transmembrane potential. This lectin-mediated cell death was inhibited by the pan caspase-inhibitor zVAD. In addition, cleavage of caspases 8, 9, and 3 was observed in Morniga-G-treated Jurkat cells whereas Jurkat cell lines that are deficient in caspase 8–10, caspase 9, or FADD, survived to the lectin-mediated toxicity. Furthermore, in the presence of TRAIL- or DR5-blocking mononoclonal antibodies, Jurkat cells became resistant to Morniga-G, suggesting that the lectin triggers cell death via the TRAIL/DR5 pathway. In silico computer simulations suggest that Morniga-G might facilitate both the DR5 dimerization and the building of TRAIL/DR5 complexes. Finally, upon treatment of Jurkat cells with benzyl-GalNAc, an *O*-glycosylation inhibitor, a decrease in Tn antigen expression associating with a reduced Morniga-G toxicity, was observed. Taken together, these results suggest that Morniga-G induces the cell death of Tn-positive leukemic cells via concomitant *O*-glycosylation-, caspase-, and TRAIL/DR5-dependent pathways.

## 1. Introduction

Glycosylation is known to contribute to different recognition and activation cell events but also to the cell death processes, altogether occurring during normal functioning of the cellular immune system [1,2,3,4]. Previous results demonstrated that plant lectins displaying very similar monosaccharide-binding specificity and three-dimensional structure differ in their capacity to recognize subtle alterations in the glycosylation induced by the lymphocyte activation process [5]. In this respect, two closely structurally-related Man-specific lectins, artocarpin from *Artocarpus integrifolia* [6] and Morniga-M from *Morus nigra* [7], both activate human resting T-lymphocytes but only Morniga-M induces cell death of activated T cells [5]. This observation suggests that Morniga-M, in addition to recognizing glycan structures associated to proliferative signaling pathway(s), might also interfere with glycan structures involved in the cell death signaling pathway(s).

Aberrations in the glycosylation processes have been reported to occur frequently in tumor cells. Truncation of the *O*-glycan chains at the cell surface of cancer cells result in the formation of T (Galβ1-3GalNAcα1-O-Ser/Thr, i.e., CD176) and Tn (GalNAcα1-O-Ser/Thr, i.e., CD175) antigens [8,9,10,11,12]. In healthy cells, Tn and T consist of the first and second step in the *O*-glycosylation biosynthesis pathway, respectively, just before a further elongation giving longer Gal/GalNAc-containing *O*-glycans. Both types of tumor-associated carbohydrate antigens (TACAs) have been identified in 70–90% of colon, stomach, bladder, lung, ovary, prostate, and cervix cancers whereas they have not been, or only slightly, expressed in healthy tissues and organs [9,10,12]. Morniga-G, the Gal/GalNAc-specific lectin from black mulberry, displays high affinity for both T and Tn antigens, in cell-free systems [13].

Leukemic Jurkat A3 cells, which expose a major proportion of Tn antigen at the cell surface, associated to a minor amount (17%) of more complex *O*-glycans, represent an ideal model for studying the defect in the *O*-glycosylation inititiation associated to cancer cells [14]. The *O*-glycosylation alterations occurring in Jurkat cells basically depend on the mutation of core 1 β3-Gal-T-specific molecular chaperone (Cosmc), that inhibits the C1β3Gal-T activity and the further addition of Gal to the Tn antigen [15]. We previously demonstrated that Morniga-G was specifically and quickly taken-up by Tn positive leukemia Jurkat T cells but not by Tn negative normal T lymphocytes [16]. Accordingly, Morniga-G covalently coupled to a photosensitizer was successfully used to design conjugates for anticancer phototherapy that was susceptible to specifically target the *O*-glycosylation alterations of tumor cells. An in vitro evaluation of these conjugates, used at 15–30 nM concentrations, readily improved the drug endocytosis and phototoxicity (by a factor 1000) in various tumor cells [17]. However, in our in vitro experiments, unconjugated Morniga-G displayed a toxic activity on Jurkat T cells at 1 mM concentration, irrespective of the photo-irradiation of the cells.

Being a component of the extrinsic apoptotic pathway, the Tumor Necrosis Factor (TNF) -related apoptosis-inducing ligand (TRAIL) specifically interacts with DR4 (TRAIL receptor-1) and/or DR5 (TRAIL receptor-2), to recruit the Fas-associated adaptor protein with a death domain (FADD), which binds to the procaspase-8 or procaspase-10 to create the death-inducing signaling complex (DISC). Both DR4 (CD261) and DR5 (CD262) possess four to five potential *O*-glycosylation sites, and mutations in the DR5 were previously reported to inhibit the TRAIL-mediated apoptosis, suggesting that *O*-glycosylation of the TRAIL/DR5 complex controls the sensitivity of tumor cells to the preapoptotic TRAIL ligand [18].

In the present work, the toxic effects of the Tn-specific plant lectin Morniga-G, were checked on leukemia Jurkat T cells, which present an impairment in the *O*-glycosylation, i.e., a strong expression of Tn antigen [12], and express the TRAIL receptor DR5 [19]. Our results suggest that the cancer-induced defects in DR5 *O*-glycosylation occurring in Jurkat T cells, could serve as a target for Morniga-G for inducing cell death via the TRAIL signaling pathway. 

## 2. Results

### 2.1. Morniga-G activates T, B, and NK Lymphocytes and Induces Cell Death in Tn Positive Leukemia Lymphocytes

**Figure 1:**
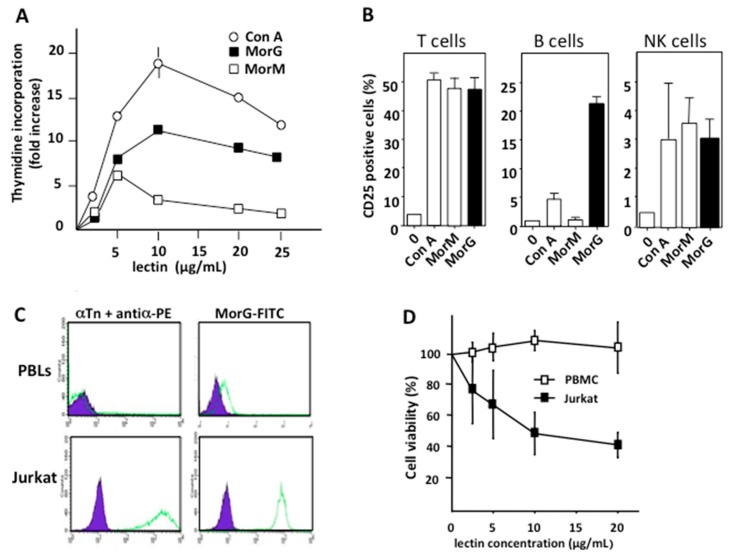
Morniga-G activates healthy human lymphocytes and induces cell death in leukemia cells. PBMCs from healthy donors were cultured for 3 days: (**A**) in the presence of increasing concentrations of Morniga-M (MorM), Morniga-G (MorG), and Con A and proliferative index (from the [^3^H]-thymidine incorporation) was calculated; and (**B**) in the presence of lectins at concentrations triggering maximal proliferation. CD25 expression was evaluated using flow cytometry, in CD3+ T lymphocytes, in CD19+ CD3- B lymphocytes, and in CD56+ CD3- NK lymphocytes. Values are means ± SD of three experiments performed with three to four different healthy donors. (**C**) Resting PBMCs and Jurkat A3 leukemic cells were incubated with anti-Tn mouse monoclonal antibody + PE-conjugated anti-mouse antibody (αTn + antiα-PE) or FITC-conjugated Morniga G (MorG-FITC) and analyzed using cytofluorimetry. Healthy peripheral lymphocytes (PBLs) were analyzed in a gate corresponding to lymphocytes as defined by the size and granularity parameters. Autofluorescence: purple histograms, dashed green histogram: fluorescent positive cells. (**D**) PBMCs of healthy donors and Jurkat A3 leukemic cells were cultured for 24 h with different concentrations of MorG, then cell viability was evaluated in 3-(4,5-dimethylthiazol)-2-5-diphenyl terazolium bromide MTT reduction assays (mean values ± SD of four independent experiments).

Plant lectins with different monosaccharide-binding specificities, e.g., the Man/Glc-specific Con A and the GlcNAc-specific pokeweed (*Phytolacca americana*) mitogen PWM, are known for a long time to readily activate resting lymphocytes. The in vitro effect of Gal/GalNAc/Tn-specific lectin Morniga-G was evaluated on resting human lymphocytes, and results were compared to Con A and Morniga-M, the Man-specific lectin from *Morus nigra*. Morniga-G induced the proliferation of 3-day-old cultured Peripheral blood mononuclear cells (PBMCs) at a concentration ranging between 2.5 and 25 μg/mL, with an optimal effect at 10 μg/mL (Figure 1A).

A similar proliferative effect was observed with Con A. In contrast, 10 μg/mL Morniga-M dramatically decreased the thymidine incorporation, compared to the rather limited decrease observed for Morniga-G and Con A. Expression of CD25 measured by cytofluorometric analysis on T, B, and NK lymphocyte populations after 3 days of cultivation in the presence of lectin concentrations yielding the higher rate of proliferation, showed that Morniga-G preferentially activated B cells, whereas both of the other lectins triggered a similar CD25 expression in T and NK cells (Figure 1B).

Compared to healthy peripheral blood lymphocytes (PBLs), Jurkat lymphoid leukemic cells highly express Tn antigen at the cell surface (Figure 1C) and, accordingly, a correlation was observed between the Tn expression level in both types of cells and the binding of Morniga-G, as shown from cytofluorometric measurements (Figure 1C). Moreover, after a 24 h-cultivation period in the presence of Morniga-G at concentrations ranging from 5 to 20 μg/mL, a substantial decrease of cellular viability was observed in Jurkat cells, whereas the growth of PBMCs was not affected (Figure 1D).

### 2.2. Morniga-G Induces Caspase-Dependent Cell Death in Tn-Positive Jurkat Cells

**Figure 2:**
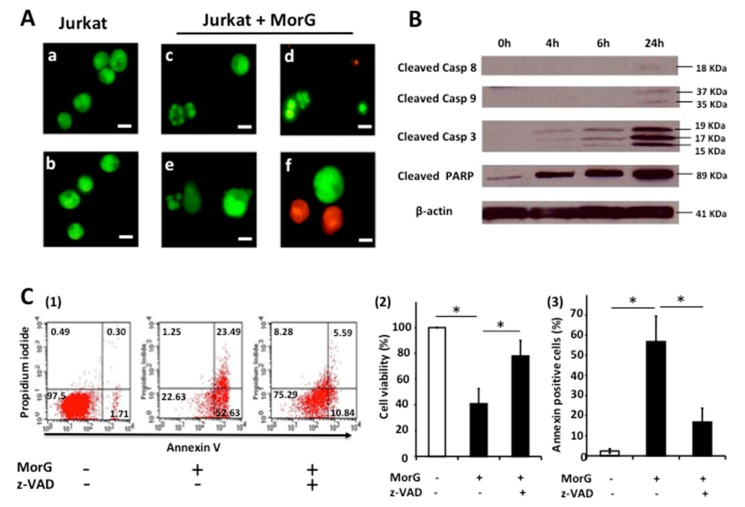
Analysis of Morniga-G-induced cell death in Jurkat leukemic cells. Jurkat A3 leukemic cells were incubated for 24 h with MorG (20 μg/mL), then analyzed using (**A**) fluorescence microscopy after Syto13 (green fluorescence) + propidium iodide staining (red fluorescence), Scale bars = 10 μm, (**B**) western blotting after staining with various anti-caspases and anti-PARP mAbs, and (**C**) cytofluorometry after Propidium iodide PI + FITC-annexin staining or MTT assays. (**A**,**B**) are representative of three identical experiments, (**A**) c and d show apoptotic cells whereas e and f show necrotic cells. (**C**) Cells were incubated with MorG in the presence of absence of z-VAD-fmk, a cell-permeant pan-caspase inhibitor: (**1**) cytofluorometric representative experiment, (**2**) MTT assays, (**3**) PI + FITC-annexin staining. Results are mean ± SD of three independent experiments, * *p* < 0.05.

The microscopic examination of the Morniga-G-treated Jurkat A3 cells after staining with Syto13 + propidium iodide, revealed the morphological changes typical for apoptosis in the majority of the cells, together with a minority of necrotic morphologies, that suggests that several cell death mechanisms are concomitantly activated after treatment of Jurkat cells with the lectin (Figure 2A).

Evaluating the cleavage of caspase-3, 8, 9, and Poly (ADP-Ribose) polymerase (PARP), in western blot experiments, revealed a cleavage of caspase-3 and PARP after 6 h of incubation in Morniga-G treated Jurkat cells, whereas the cleavage of other caspases 8 and 9 occurred later (Figure 2B). This result suggests that a caspase-3-dependent signaling pathway becomes rapidly activated upon treatment with the lectin. In addition, the Morniga-G-induced cell death was significantly inhibited in Jurkat cells previously cultured in the presence of the caspase inhibitor z-VAD (Figure 2C), suggesting Morniga-G is capable of activating signaling pathways involving different caspases to induce Jurkat cell death.

### 2.3. MorG Activates Different Steps of Extrinsic and Intrinsic Pathways of Caspase-Dependent Cell Apoptosis in Tn-Positive Jurkat Cells

**Figure 3:**
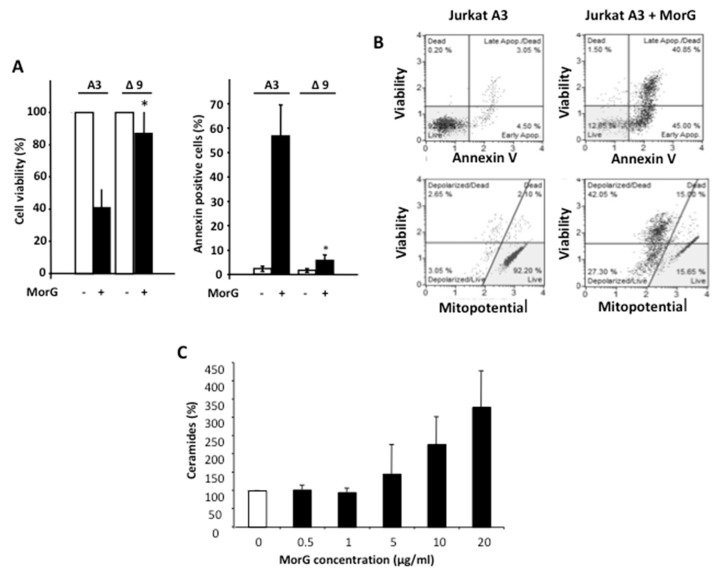
Morniga-G-induced cell death involves mitochondria, ceramides and caspase 9 (intrinsic pathway). Jurkat A3 leukemia cells were incubated for 24 h wit MorG (20 μg/mL). (**A**) MorG-mediated toxicity was evaluated by MTT assay (% of viable cells) or by annexin/PI IP and cytofluorometry (% of annexin-positive cells), in Jurkat parental leukemia cells (A3) and in caspase 9-deficient Jurkat cells (Δ9) treated with MorG (20 μg/mL). Results are mean ± SD of three independent experiments, * *p* < 0.05. (**B**) Apoptosis and mitochondrial membrane potential (mitopotential), representative of two duplicate experiments, were analyzed using cytofluorometry in Jurkat A3 cells. (**C**) Total ceramide content measured in Morniga-G treated Jurkat A3 cells. Results are mean ± SD of three independent experiments.

To check the involvement of caspase-9 in Morniga-G-induced cell death, experiments were carried out with Δ9 Jurkat cells, a cell line characterized by a genetic deficiency in caspase-9. The absence of caspase-9 readily protected the leukemia Δ9 Jurkat cells from Morniga-G-induced cell death (Figure 3A). In addition, an evaluation of the membrane potential of the mitochondria by cytofluorimetry, showed that death of the Jurkat A3 cells was accompanied by a reversal in the mitochondrial membrane potential (Figure 3B). Finally, the amount of ceramides produced in Jurkat cells as an effect of Morniga-G treatment exhibited a marked increase in these molecules, which are known to participate in the activation of the intrinsic pathway of the caspase-induced cell apoptosis (Figure 3C).

**Figure 4:**
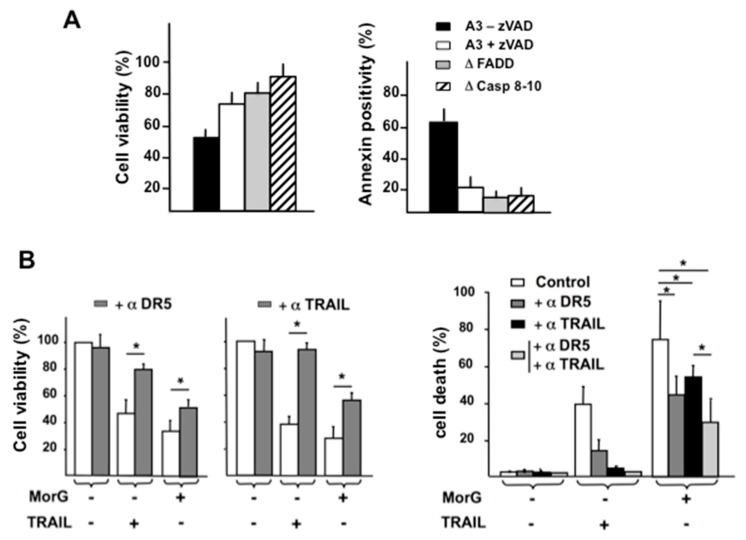
Morniga-G-induced cell death involves caspase-dependent extrinsic pathway. (**A**) Jurkat leukemic cells (A3) with or without zVAD, FADD-deficient Jurkat cells (Δ FADD), and Caspases 8- and 10-deficient Jurkat cells (Δ casp 8–10) were cultured for 24 h with or without Morniga-G (20 μg/mL). Cytotoxicity was evaluated using an MTT assay (cell viability in percentage of controls without MorG, mean ± SD of four independent experiments, * *p* < 0.05) or using annexin/IP and cytofluorometry (MorG-induced cell death, i.e., annexin positivity after subtraction of cell death percentage in control cells without MorG, mean ± SD of 3 independent experiments). (**B**) Jurkat A3 leukemic cells were cultured for 24 h with or without Morniga-G (20 μg/mL) or TRAIL cytokine (50 ng/mL), and with or without DR5 (αDR5) or TRAIL (αTRAIL) blocking monoclonal antibodies. Cytotoxicity was evaluated using an MTT assay (left panel, % of viable cells, mean ± SD of four independent experiments, * *p* < 0.05) or using annexin/IP and a cytofluorometry assay (right panel, cell death percentage, mean ± SD of three independent experiments, * *p* < 0.05).

Similarly, double-deficient cells for caspase 8 and 10, and FADD-deficient Jurkat cells, were cultured in the presence of 20 μg/mL of Morniga-G for 24 h. Caspase inhibitor zVAD was added in non-deficient Jurkat A3 cells, as a cell death inhibitory control. In these experimental conditions, as previously reported, Jurkat cells were protected against MorG-induced cell death via zVAD addition, whereas the absence of FADD or caspases 8/10 had also a strong protective effect on cell viability (Figure 4A, left). Evaluating cell death using cytofluorometric analysis suggested, however, that Morniga-G might induce cell death via FADD- and caspases 8,10- independent pathways, in a minor proportion of cells (Figure 4A, right).

Since FADD is involved in death receptor-mediated pathways of cell apoptosis and necroptosis triggered by cytokines like TRAIL, TNF, or FasL [20], cytotoxicity experiments were performed in the presence of Morniga-G and compared to TRAIL-mediated toxic effects. Jurkat cells are known to be TRAIL sensitive and express DR5, the TRAIL-receptor 2 [19]. As expected, both Morniga-G and TRAIL had cytotoxic effects on Jurkat A3 cells, as measured after a 24-h culture (Figure 4B). The TRAIL-mediated cell death was almost completely inhibited when blocking monoclonal antibodies against DR5 or TRAIL were added separately to the cell cultures.

However, addition of the blocking monoclonal antibodies only partially protected the Morniga-G-treated cells (Figure 4B, left). Finally, the simultaneous addition of both blocking antibodies resulted in a complete inhibition of TRAIL-induced cell death, whereas Morniga-G-treated cells still remained only partially protected (Figure 4B, right). These results suggest that Morniga-G induces cell death via, at least in part, an activation of the TRAIL/DR5 signaling pathway.

### 2.4. The O-Glycosylation at the Leukemic Cell Surface Is Involved in Morniga-G Induced Cell Death

**Figure 5:**
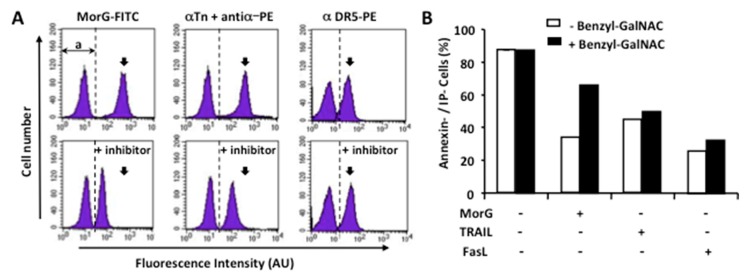
Morniga-G-induced cell death depends on *O*-glycosylation. Jurkat A3 leukemic cells were cultured with or without benzyl-GalNAc (8 mM), an *O*-glycosylation inhibitor. Subsequently, the cells were analyzed: (**A**) using cytofluorometry after staining with FITC-conjugated MorG (MorG-FITC), anti-Tn mouse monoclonal antibody + Phycoerythrin PE-conjugated anti-mouse antibody (αTn + antiα-PE) or PE-conjugated anti-DR5 monoclonal antibody (α DR5-PE). Histograms overlay, **a**: auto-fluorescence histogram of Jurkat cells without staining on the left of vertical dotted line and stained cell histogram on the right, black arrow: mean fluorescence after staining and without inhibitor-treatment, representative of three independent experiments; (**B**) cytofluorometry after annexin/IP staining. Cell viability is shown as percentage of annexin- and IP-negative cells after a 24 h culture in presence of MorG (20 μg/mL), TRAIL (50 ng/mL), or FasL (50 ng/mL). Mean of two independent experiments.

Owing to the previously observed correlation between the binding of Morniga-G to Jurkat cells and their Tn-expression level (Figure 1), the role of surface-exposed *O*-glycans in the Morniga-G cytotoxicity was further investigated. Cytofluorometric experiments aimed at evaluating the effects of benzyl-GalNAc, an *O*-glycosylation inhibitor, on both the DR5/Tn expression and the Morniga-G-induced Jurkat cell death, showed stable expression of DR5 on the inhibitor-treated Jurkat cells (Figure 5A). Conversely, a decrease in both the Tn expression and the binding of Morniga-G at the cell surface was observed in the inhibitor-treated Jurkat cells (Figure 5A). In addition, those cells expressing low levels of Tn antigen exhibited enhanced resistance to the Morniga-G-induced cytotoxicity, most probably as a consequence of the inhibition of Tn expression. In contrast, partial inhibition of *O*-glycosylation did not cause a decrease in cell death, neither in TRAIL-treated nor in FasL-treated Jurkat cells (Figure 5B). Taken together, these results suggest that Morniga-G-induced cell death in Jurkat cells is associated with *O*-glycosylation and, most probably, depends on the level of expression of Tn antigen at the cell surface.

**Figure 6:**
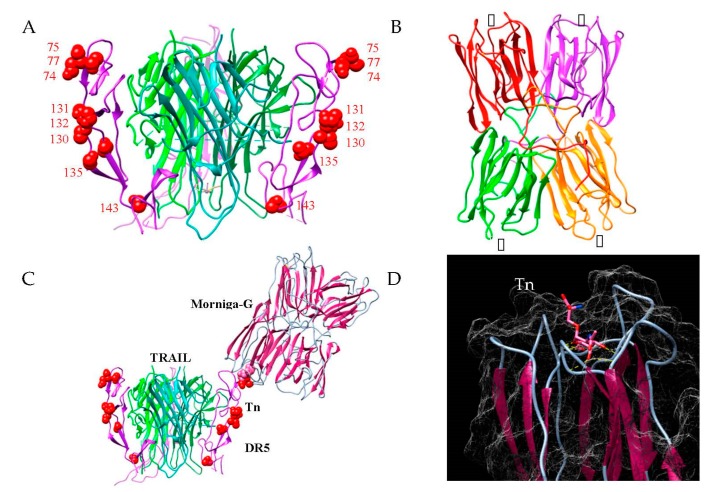
Morniga-G might interact with Tn antigens presented by DR5 receptor. (**A**) Mapping of *O*-glycosylation sites (Ser and Thr residues) on the crystallographic complex of the TRAIL trimer (green colors) with DR5 (purple), PDB code 1DU3 [18]. Ser residues 74, 75, 77, and Thr residues 130, 131, 132, 135, and 143 are represented in red spheres. (**B**) Three-dimensional model built for Morniga-G, made of four protomers, each containing a Tn-specific binding site (▯). (**C**) Cartoon showing the interaction between Morniga-G and the DR5 receptor complexed to its natural TRAIL ligand, via the recognition of Tn antigen located on Ser residue 75. (**D**) Docking of Tn antigen to the Tn-specific binding site of Morniga-G. Yellow dashed lines indicate the network of hydrogen bonds connecting Tn antigen to the lectin.

**Figure 7:**
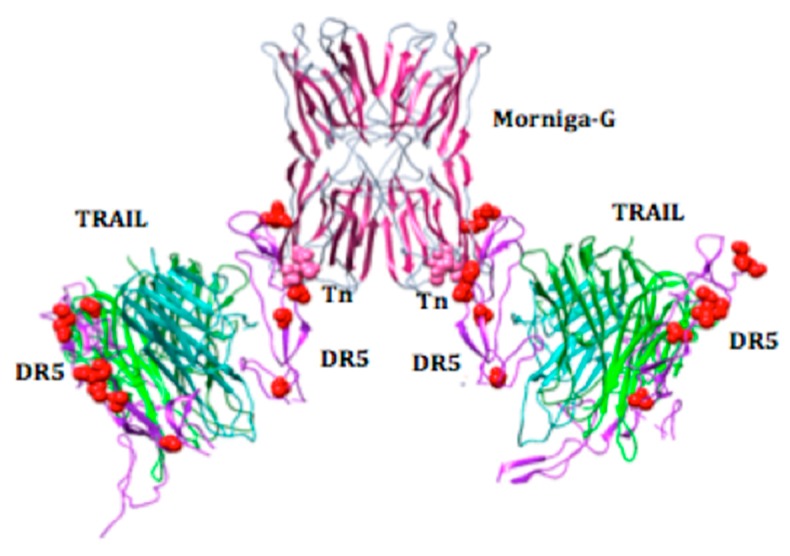
Morniga-G could facilitate the aggregation of Trail-DR5 complexes. Simulation drawn from docking experiments, showing how Morniga-G could facilitate the dimerization/aggregation of the TRAIL/DR5 complexes (TRAIL trimers in green/DR5 in purple) via the specific recognition of Tn antigen associated to Thr131 (colored pink) of DR5 monomers. Similarly, the lectin could facilitate the aggregation of the TRAIL/DR5 complexes via specific interactions with Tn antigen associated to Thr 130 of DR5.

As previously described (Protein Data Bank code 1DU3), the extracellular domain of the TRAIL-DR5 complex consists of three DR5 monomers tightly associated by non-covalent bonds to the TRAIL homotrimer, to form a symmetric ligand-receptor complex [20,21,22]. Since DR5 contains several putative sites of *O*-glycosylation—namely Ser74, Ser75, and Ser77, and Thr130, Thr131, Thr132, Thr135, and Thr143, respectively (Figure 6A)—it is tempting to speculate that Tn antigen molecules probably expressed at Ser and/or Thr residues of DR5 can serve as possible ligands for the four Tn-specific binding sites of Morniga-G (Figure 6B,D). In order to reinforce this hypothesis, docking experiments performed between the three-dimensional model built for Morniga-G and the crystallographic complex TRAIL-DR5 (PDB code 1DU3), suggested a possible interaction between Morniga-G and several Tn-containing Ser or Thr residues of DR5 (Figure 6C). Moreover, these Tn-mediated interactions between Morniga-G and the TRAIL-DR5 complexes, could facilitate in some way the dimerization and oligomerization process occurring between the TRAIL-DR5 complexes at the surface of Jurkat cells. In this respect, the well-exposed Tn antigens associated to both Thr130 and Thr131 residues, could play a key role in the binding of Morniga-G to create transient molecular bridges important to facilitate the dimerization/oligomerization of the TRAIL-DR5 complexes (Figure 7). However, while this hypothesis is attractive, it remains fully speculative and, obviously, additional experiments will be necessary in future either rebut or confirm its reality.

## 3. Discussion

As shown from our results, Morniga-G, a Gal/GalNAc- and Tn-specific lectin, readily activated healthy lymphocytes to trigger cell proliferation and associated CD25 expression (Figure 1A,B), where both events are considered as the first step in the lymphoid activation [23]. However, resting lymphocytes were characterized as Tn-negative cells and only moderately interacted with Morniga-G, compared to Tn-positive Jurkat cells, which heavily interacted with the lectin. Accordingly, the mitogen activity of Morniga-G-binding on resting lymphocytes most probably relies on the presence of Gal/GalNac residues in some activator glycoproteins and glycolipids, which are sufficiently accessible at the cell surface to interact with the lectin, as previously reported for other lectins [24].

Both Man-specific lectins Con A and Morniga M triggered the activation of T and NK lymphocytes. Interestingly, Morniga-G strongly activated B-lymphocytes, similar to the pokeweed mitogen (PWM), which also activates B-lymphocytes and T-lymphocytes as well [25]. However, human peripheral blood B-lymphocytes cultured with PWM in the absence of other cells were unable to proliferate, and their activation was dependent on the presence of accompanying T cells and monocytes [25]. Since (1) monocytes account for 10–20% of the PBMCs used in our experiments, and (2) Morniga-G binds equally well to both monocytes and lymphocytes (results not shown), activation of B lymphocytes by Morniga-G most probably depends on cooperation between previously lectin-activated T cells and monocytes, e.g., by induction of IL-6 secretion, a B cell-activating, and proliferative cytokine. Finally, the Gal/GalNAc-specific lectin Morniga-G, can activate healthy T and NK cells, similar to the Man/Glc-specific Con A, and also B cells, similarly to the GlcNAc specific PWM. In agreement with previous observations, the present results suggest that lectins with different monosaccharide-binding specificities may recognize subtle differences in the oligosaccharide patterns occurring at the surface of activation molecules, to trigger the effective activation of lymphocytes. As with Con A, PHA (Phytohemagglutinin from *Phaseolus vulgaris*), or PWM, the activation by Morniga-G probably involves the specific recognition of oligosaccharide patterns carried by CD molecules involved in lymphoid activation, e.g., CD3, CD45, or CD79 molecules.

It is well established that carcinogenesis is associated with changes in the cell glycome, such as the expression of an aberrant *O*-glycosylation [8,9,10,11,12]. These modifications in the *O*-glycosylation are most likely involved in various tumoral processes, e.g., the epithelial to mesenchymal transition (EMT) and the formation of metastasis [26,27,28]. The occurrence of truncated *O*-glycan chains in the form of T or Tn antigens, is one of the modifications frequently encountered at the surface of tumor cells [9,10,11,12]. A number of Gal/GalNAc-binding lectins from plants and fungi can specifically interact with *O*-glycans occurring at the cell surface, and potentially induce cell function alterations. In addition, a few Gal/GalNAc-specific lectins recognize T and Tn antigens with a high affinity, e.g., PNA from peanuts, jacalin from Jackfruit, ricin from castor beans, and Morniga-G [13]. Some of these lectins displayed some cytotoxicity toward tumor cells in vitro, especially the lectins with a ribosome inactivating domain or type 2 ribosome-inactivating proteins (RIPs). Obviously, lectins with RIP-activity, like ricin, abrin, or ebulin, are highly toxic for both healthy and cancer cells, mainly by inhibiting their protein synthesis. Consequently, these lectins could be used as toxic compounds in targeted cancer therapy, e.g., for immunotoxin manufacturing [29,30,31,32]. Other Gal/GalNAc-specific plant lectins like PNA (Peanut agglutinin), SBL (Soybean lectin), and VVLB4 *(Vicia villosa* isolectin B4), can also induce cell death but their toxicity is at least 1000 times lower than that of ricin and abrin [13]. Moreover, they trigger autophagy, necrosis, or apoptosis in different human carcinoma cells, and these lectin-induced cell deaths are associated with various biochemical signals, such as the generation of Reactive oxygen species ROS or caspase activation [33,34,35,36].

In Jurkat cells, activation of caspases 8, 9, and 3, and cleavage of PARP accompanying the Morniga-G treatment, suggest an involvement of both intrinsic and extrinsic pathways in the Morniga-G-induced cell death. First, Morniga-G elicits both a caspase 9-dependent cell death, the reversal of mitochondrial membrane potential, and an increase in total ceramides, which is in agreement with an activation of an intracellular intrinsic pathway (Figure 3). Furthermore, we have previously shown that FITC-Morniga-G was quickly endocytosed by Jurkat cells (<5 min), making the lectin readily available for possible intracellular effects [16]. Second, the Morniga-G-induced cytotoxicity was inhibited in FADD- and caspase 8–10-deficient Jurkat cells, suggesting an activation of the death receptor pathways by the lectin using FADD and caspase 8. Jurkat cells are known to express TNF, FasL, and TRAIL receptors and to respond positively to both cytokines. Among these three types of receptors, only DR5 TRAIL-receptors possess putative sites for *O*-glycosylation, namely well-exposed Ser and Thr residues [18,20]. In addition, Jurkat cells are known to express DR5 but not DR4 [19]. Accordingly, the resistance of leukemic cells to Morniga-G-mediated cytotoxicity after treatment with anti-DR5 and anti-TRAIL blocking mAbs, strongly suggests an involvement of the TRAIL/DR5 pathway in cell death. However, this protection was only partial, as compared to TRAIL-mediated cytotoxicity (Figure 4B), suggesting that other mechanisms might be triggered after treatment of Jurkat cells with the lectin. Thus, Morniga-G could interact with T/Tn antigens or other Gal/GalNac residues on TNF receptor I (CD120a) or FasL (CD95), or other glycoproteins distinct from the cell death receptors, but triggering a rapid release of TNF, FasL, or TRAIL. In this respect, it was previously shown that a treatment of Jurkat T cells with PHA or an anti-CD59 mAb, induces the release of TRAIL, TNF, and FasL cytokines, to mediate the so-called “activation-induced cell death” process [37]. In addition, some activating glycoproteins are known to be sufficiently *O*-glycosylated to initiate cell death in proliferating or activated lymphocytes, e.g., CD45 and CD7 [2,38,39], both expressed in Jurkat T cells. Another interesting pathway, the *O*-glycosylation of CD7, has also been described as a target for human galectin-1, a β-galactoside binding lectin. The binding of galectin-1 can induce cell death in both T-lymphoma cells and healthy activated T cells [40].

Both the binding and endocytosis of Morniga-G in Jurkat cells is inhibited by adding inhibitory sugars, e.g., Gal, GalNAc, or Tn antigen, or *O*-glycosylated glycoproteins, e.g., the bovine serum mucin, prior to the lectin [16,17]. Accordingly, our results suggest that Morniga-G-mediated toxicity depends on some *O*-glycan recognition, most probably Tn antigen recognition. From our results, it is tempting to speculate that Tn recognition by the lectin can elicit lymphocyte cell death by cross-linking of non-FADD dependent receptors like CD45 or CD7 [2,38,39]. However, our results suggest that TRAIL/DR5 pathway plays a major role in the Morniga-G-induced cell death of Jurkat cells. As hypothesized by in silico docking experiments, the interaction of Morniga-G with Tn antigen haptens located on DR5 in the TRAIL/DR5 complex, could favor the building of TRAIL/DR5 lattices or increase their efficiency in the TRAIL-mediated apoptotic cell death [20,21,22]. 

In conclusion, evidently it is difficult to predict that the Morniga-G lectin will be useful in the cancer human treatment from these in vitro data alone. However, the present results show that non-RIP lectins able to specifically recognize aberrations in *O*-glycosylation, could serve as cytotoxic molecules against cancer cells. In addition, these lectins offer some exciting new carriers for improving the drug targeting in cancer treatment [13].

## 4. Materials and Methods

### 4.1. Cell Lines and Reagents

Parental Jurkat A3 leukemia cell line (from ATCC, Manassas, VA, U.S.), FADD-deficient Jurkat cells (Δ FADD, from Dr. J. Blenis, Boston, MA, USA), caspase 9-deficient Jurkat cells (Δ 9, from Dr. K. Shulze-Osthoff, Düsseldorf, Germany), and Caspase 8- and 10-doubly deficient Jurkat cells (Δ casp 8–10, from our laboratory [41]), were cultured in RPMI medium containing 10% FCS. Peripheral blood mononuclear cells (PBMC) were separated using Ficoll-Paque Plus (Amersham Biosciences, Piscataway, NJ, USA) density gradient and cultured in RPMI containing 10% FCS.

Concanavalin A (Con A) was purchased from Vector Laboratories (Burlingame, CA, USA). Morniga-G (MorG) and Morniga-M (MorM) were purified from the bark of a black mulberry tree (*Morus nigra*), as previously described [7]. Morniga-G was labelled with FITC (Acros Organics, Fisher Scientific, Illkirch, France) as previously described [16].

The pan-caspases inhibitor z-VAD(OMe)-fmk was purchased from Bachem (Voisins-Le-Bretonneux, France) and benzyl-2-acetamido-2-desoxy-α-d-galactopyranoside (benzyl-GalNAc) from Merck (Darmstadt, Deutschland) city, state abbreviation if USA or Canada, country). TRAIL was purchased from PeproTech (Neuilly-Sur-Seine, France)(city, state abbreviation if USA or Canada, country) and FasL was produced from Neuro2A cells transfected with a plasmid encoding FasL [41].

Anti-CD175 (anti-Tn) mouse monoclonal antibody (mAb) was purchased from Acris Antibodies GmbH (Herford, Germany) and fluorochrome-conjugated rat anti-mouse polyclonal antibodies from Thermo scientific (1 μg/mL), and PE-conjugated anti-human DR5 from Abcam (Cambridge, UK). Human recombinant blocking monoclonal antibodies anti-DR5 (1 µg/mL) and anti-TRAIL (1 µg/mL) were purchased from Diaclone (Besançon, France), and anti-Fas (1 μg/mL) from Bender MedSystems (Vienna, Austria).

Allophycocyanin APC-conjugated anti-CD19 mAb was purchased from Dako (Glostrup, Denmark). APC-conjugated anti-CD56, FITC-conjugated anti-CD25 mAbs were from Pharmingen (San Diego, CA, USA). Different fluorochrome-labelled anti-CD3 or anti-CD14 mAbs were from Becton Dickinson (Franklin Lakes, NJ, USA).

### 4.2. Human Peripheral Blood Cell Culture, Proliferation, and Activation Assays

PBMCs were isolated from healthy donors using Ficoll-Paque Plus (Amersham Biosciences, Piscataway, NJ, USA) density gradient centrifugation at 2000 rpm for 20 min. Routinely in PBMC, lymphocytes were in the range of 80–90%, whereas monocytes (CD14-positive cells) ranged from 10–20%, as evaluated using cytofluorometry. The cells (10^6^ cells/mL) were cultured in triplicate in U bottom 96-well plates (100 mL/well) or in 24-well plates (1 mL/well) in RPMI 1640 supplemented with 10% fetal calf serum (Gibco, Cergy-Pontoise, France). For proliferation assays, Morniga-M, Morniga-G and Con A, were added at different concentrations ranging from 2.5 to 25 μg/mL. Subsequently, cells were incubated for 72 h at 37 °C in a 5% CO_2_ humidified atmosphere, and [^3^H]-Thymidine (5µCi/well) (ICN, Orsay, France) was added for the final 16 h of incubation. The content of the plates was harvested onto a glass fibre filter using a 96 well automatic cell harvester (Harvester 96 Tomtec, Wallac-EG&G instruments, Evry, France) and the [^3^H]-Thymidine incorporation was measured by liquid scintillation counting (Wallac, PerkinElmer, Waltham, CA, USA). Proliferation indexes of triplicates were calculated from the ratio mean counts per min (cpm) of experimental assay/negative control. Lectin-triggered lymphocyte activation was assessed after incubation for 72 h in the presence of lectins at concentrations yielding maximum proliferation. These concentrations were 5 μg/mL (0.075 mM) for Morniga-M, and 10 μg/mL for Con A (0.1 mM) and Morniga-G (0.14 mM). Naive or lectin-activated lymphocytes were washed with Phosphate Buffer Saline PBS (pH 7.4), pelleted by centrifugation at 1400 rpm for 5 min, and then stained at 4 °C. Specific activation of lymphocyte subsets (T cells, B cells, and NK cells) was checked after 72 h cell culture (as described above) by adding a mixture of labelled antibodies (PerCP anti-CD3,/FITC anti-CD25, APC anti-CD19/FITC anti-CD25 or APC anti-CD56/FITC anti-CD25), for 30 min in the dark. Then, cells were washed with PBS (pH 7.4) and re-suspended in 300 μL of PBS (pH 7.4, 1% PFA). Twin CD receptor expression was monitored by a FACSCan flow cytometer (BD Biosciences, Franklin Lakes, New Jersey, USA) city, state abbreviation if USA or Canada, country), in a gate corresponding to lymphocytes as defined by the size and granularity parameters.

### 4.3. Cell Surface Binding Experiments

Cells (10^6^/mL) were incubated with FITC-Lectin (0.25 μg/mL), anti-Tn antibody (1 μg/mL), or PE-conjugated anti-DR5 antibody (1 μg/mL) for 30 min at 4 °C in PBS. Cells were washed and stained with PE-conjugated secondary antibody for 30 min at 4 °C in PBS. After washing, cells were analyzed using flow cytofluorometry. Concerning *O*-glycosylation inhibition assays, benzyl-GalNAc was added in the culture medium for 72 h at 8 mM before performing cell surface binding experiments or cytotoxicity assays.

### 4.4. Lectin-Mediated Cytotoxicity Assay and Cell Death Evaluation

Briefly, Jurkat leukemic T cells (A3, Δ 9, Δ FADD, or Δ casp 8–10) were exposed to Morniga-G (20 μg/mL) for 24 h in RPMI-SVF 10% at 37 °C/5% CO_2_ humidified atmosphere. In some experiments, the parental Jurkat A3 leukemia cells were pre-treated (2 h) or not with pan-caspases inhibitor zVAD (20 μg/mL), or pre-treated (72 h) with the *O*-glycosylation inhibitor benzyl-GalNAc (8 mM) then co-incubated with Morniga-G. For blocking assays, anti-DR5 and/or anti-TRAIL neutralizing antibodies were used. TRAIL (50 ng/mL) or mouse FasL (50 ng/mL) were used as control cell death assays. Cell death was then estimated by: (i) 3-(4,5-dimethylthiazol)-2-5-diphenyl terazolium bromide (MTT) reduction method (Euromedex, Souffelweyersheim, France), and (ii) flow cytofluorometry analysis using annexin V-FITC/propidium iodide (PI) staining (AbCys, Paris, France). The cell death was determined, after debris exclusion.

Cell death morphology was analyzed using Syto 13 (Molecular Probes, Eugene, OR, USA) and PI staining with a fluorescence-equipped microscope (Olympus, SELI, Toulouse, France) as previously described [41].

For mitochondrial analysis, cells were cultivated with lectins for 24 h and cell viability and mitochondrial membrane potential (mitopotential) were analyzed by Muse Annexin V and Dead Cell Assay kit (#MCH100105) and Muse MitoPotential Kit (#MCH100110), using Muse Cells Analyzer (Merckmillipore) (Merck, Darmstadt, Deutschland).

### 4.5. SDS-PAGE and Western Blot Analysis

Western blots were performed as described elsewhere [41]. Briefly, Jurkat A3 cells were cultured for different incubation periods (4, 6, or 24 h) in the presence or absence of the lectin. Protein extracts (20 µg) were separated on SDS-PAGE (sodium dodecyl sulfate-polyacrylamide gel electrophoresis) and blotted on nitrocellulose membranes. The blots were analyzed using anti-cleaved PARP, anti-cleaved Caspase-9, -3, and -8 (Cell Signaling Technology, Danvers, MA, USA) or anti-ß Actin (Sigma, Saint-Quentin Fallavier, France) antibodies.

### 4.6. Ceramide Measurement

Ceramide mass was determined as previously reported [42], using recombinant DAG kinase (kind gift from Drs. D Perry and YA Hannun; Charleston, NC, USA). Briefly, lipids were extracted and solubilized before DAG kinase assay. Lipid content was then separated by thin layer chromatography, and ceramide-1-^32^Phosphate was quantified by phosphor imaging.

### 4.7. In Silico Molecular Modeling and Docking Experiments

Homology modeling of Morniga-G was performed with the YASARA Structure program [43], using the X-ray coordinates of the closely related Man-specific lectin Morniga-M (PDB code 1XXR) [44], as a template. Although the homotetrameric model built up for Morniga-G exhibited a three-dimensional structure closely similar to that of Morniga-M, it differs from the template by the single-chain structure of the four protomers forming the Morniga-G homotetramer. PROCHECK [45], ANOLEA [46], and the calculated QMEAN score [47,48], were used to assess the geometric and thermodynamic qualities of the three-dimensional model. Using ANOLEA to evaluate the model, 18 residues (out of 155) of the model exhibited an energy over the threshold value. Most of these residues were mainly located at the N-terminal end in the four protomers forming the homotetrameric structure of Morniga-G. However, the calculated QMEAN4 score gave an acceptable value of −0.85.

Docking of Tn antigen to the monosaccharide-binding site Morniga-G was performed with the YASARA structure program. Some docking experiments were performed at the SwissDock web server (http://www.swissdock.ch) [49,50] as a control for our docking experiments. Molecular cartoons were drawn with Chimera [51].

### 4.8. Statistical Analyses

Results are expressed as the means ± SD of data obtained from at least three independent experiments. Statistical significance was determined by means of Student’s *t*-test. *p* < 0.05 was considered significant.

## Abbreviations

Cosmc	Core 1 b3-Gal-T-specific molecular chaperone
DISC	Death-inducing signaling complex
DR4	Death receptor 4
DR5	Death receptor 5
FADD	Fas-associated adaptor protein with a death domain
FCS	Fetal calf serum
FITC	Fluorescein isothiocyanate
mAb	Monnoclonal antibody
Morniga-G	Gal-specific black mulberry (*Morus nigra*) lectin
MTT	3-(4,5-dimethylthiazol-2-yl)-2,5-diphenyltetrazolium bromide
NK	Natural killer
PARP	Poly (ADP-Ribose) polymerase
PBL	Peripheral blood lymphocytes
PBMC	Peripheral blood mononuclear cells
PE	Phycoerythrin
PI	Propidium iodide
PS	Phosphatidylserine
T antigen	Galb1-3GalNAca1-O-Ser/Thr, i.e., CD176
Tn antigen	GalNAca1-O-Ser/Thr, i.e., CD175
TACA	Tumor-associated carbohydrate antigens
TNF	Tumor necrosis factor
TRAIL	Tumor-necrosis-factor related apoptosis inducing ligand
zVAD-fmk	*N*-Benzyloxycarbonyl-Val-Ala-Asp(O-Me) fluoromethyl ketone
